# Leveraging Artificial Intelligence for Advancements in Liquid Dosage Formulations in the Pharmaceutical Industry

**DOI:** 10.1007/s43441-025-00823-w

**Published:** 2025-06-25

**Authors:** D. Nithyanantham, Akhil Nair, Usha Y. Nayak

**Affiliations:** Center for Drug Delivery Technologies, Department of Pharmaceutics, Manipal College of Pharmaceutical Sciences, Manipal Academy of Higher Education, Manipal, Karnataka 576104 India

**Keywords:** Artificial intelligence, Pharma 5.0, Manufacturing, Fluid flow, Process analytical technology

## Abstract

Formulation strategies are vital in drug development, but trial-and-error optimization of critical parameters is inefficient. Artificial Intelligence (AI) addresses this by enabling data-driven decision-making. AI has emerged as a pivotal tool in drug discovery, formulation, and development. This manuscript explores the significant applications of AI in various aspects of liquid dosage form manufacturing, aligned with Industry 5.0 principles. This integration seeks to enhance productivity, monitor and modify development processes, enable human-centric automation, improve product quality, and optimize industry workflows. Essential steps in developmental design include solvent selection, drug-excipient compatibility, crystallinity, salt formation, permeability, and stability. This technological approach facilitates rapid decision-making and ensures batch-to-batch consistency by investigating major attrition rates in manufacturing and discovery. We highlight the utilization of AI tools and techniques alongside Process Analytical Technology (PAT) for real-time applications in the production of parenteral solutions, suspensions, emulsions, and other liquid forms. A comprehensive review of the current state of AI in common manufacturing steps involved in liquid dosage formulation development is provided, identifying potential research gaps. We discuss ongoing challenges, propose realistic strategies to address these challenges and outline future research directions.

**Graphical Abstract:**

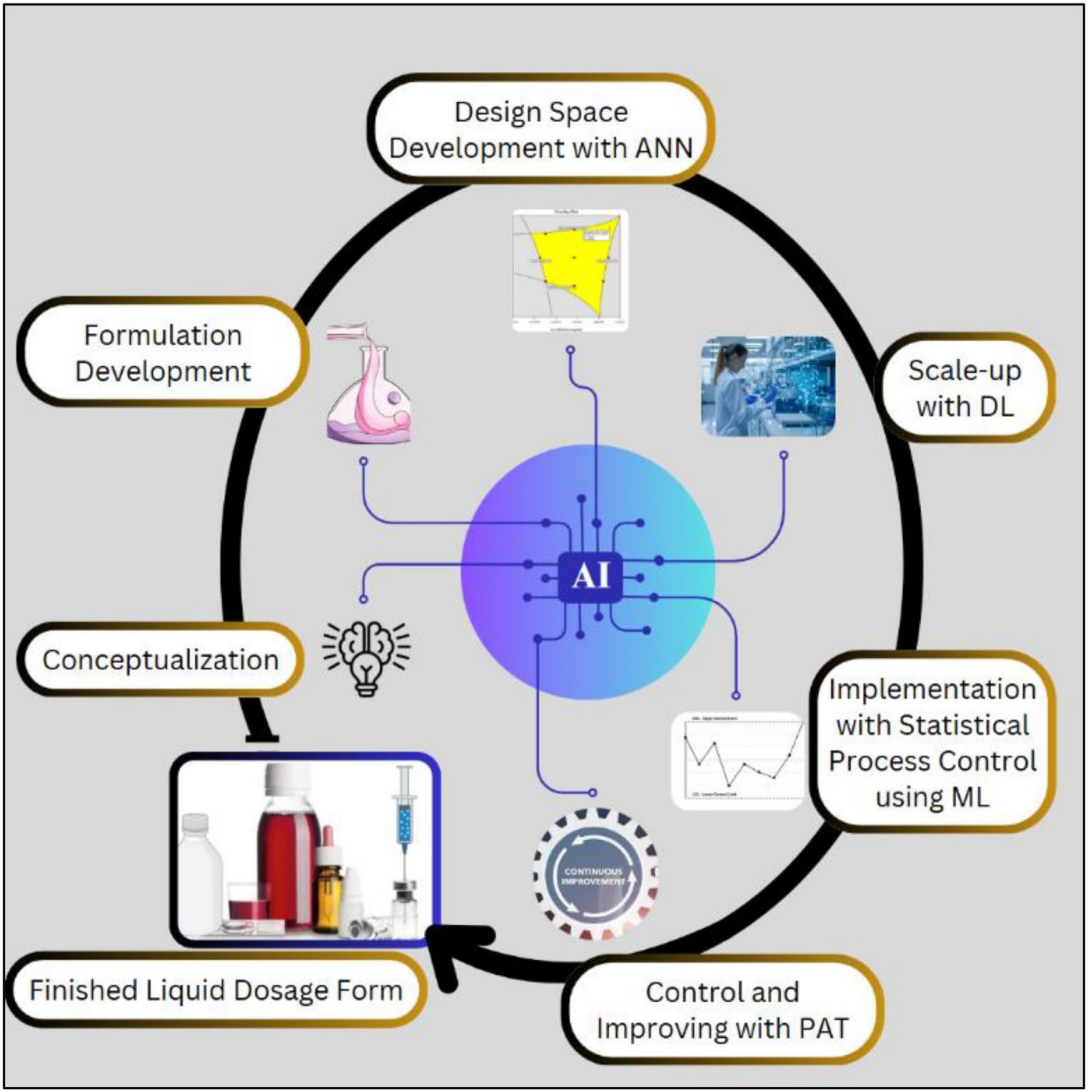

## Introduction

**Fig. 1:**
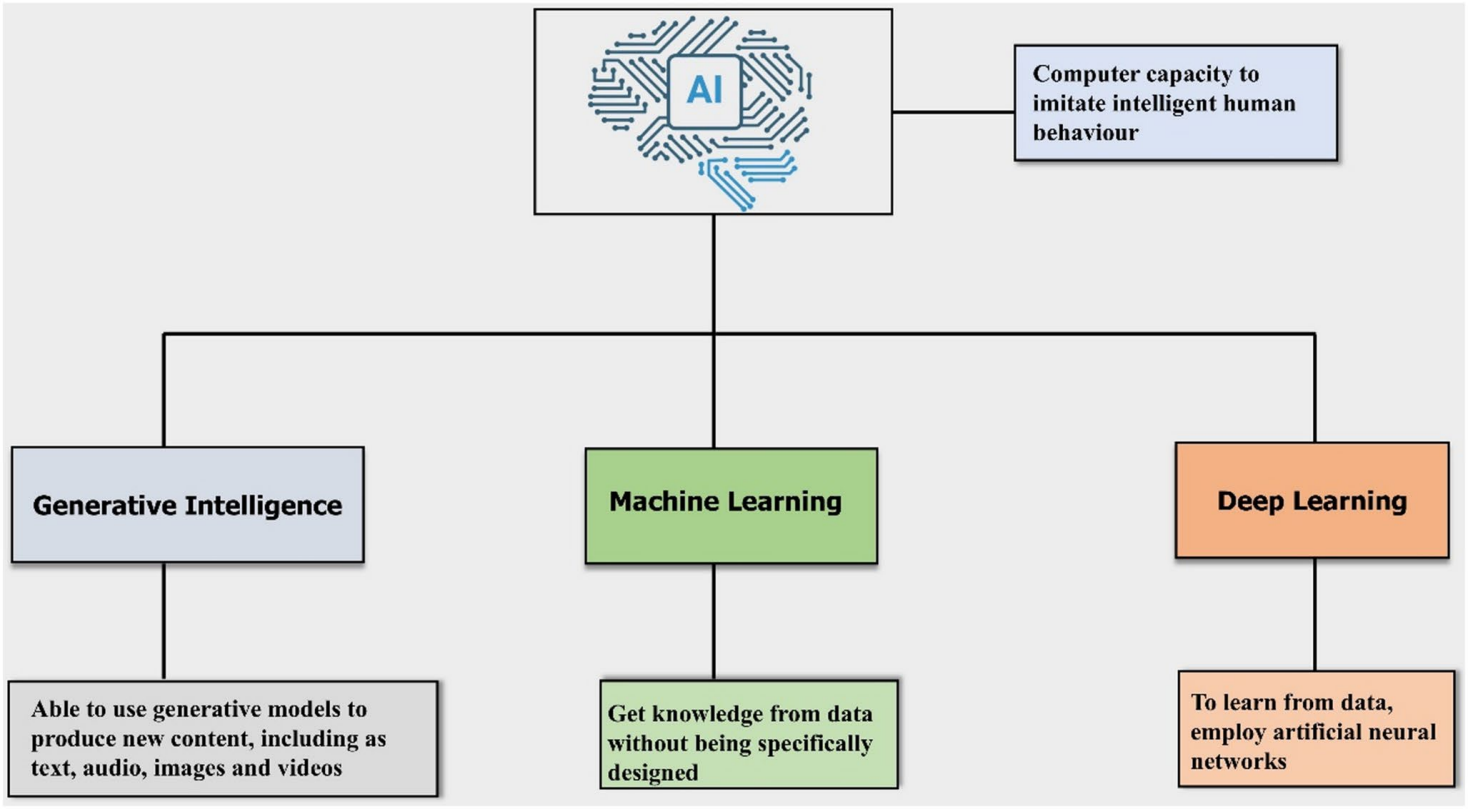
Schematic representation of Artificial intelligence (AI) and its subsets

Artificial Intelligence (AI) is transforming drug product development through its ability to convert scientific knowledge into new drug products. AI technologies are utilized in various stages of drug development pipeline by integrating diverse data sources to develop intelligent and efficient methods. This boosts the success rates by enabling faster, accurate and more efficient drug discovery and development [1]. Machine Learning (ML), a subset of AI focused on algorithms that enable the system to learn and make predictions or decisions based on data. It includes supervised learning (learning from labeled data), unsupervised leaning (finding patterns in unlabeled data), and reinforcement learning (learning through trial and error with rewards). Artificial Neural Networks (ANNs), a sophisticated method within the realm of deep learning (DL) and a deeper subset of machine learning (ML), have emerged as versatile tools for data processing and drug product development **(**Fig. 1**)**. These networks leverage advanced neural architectures, inspired by the functioning of biological neurons, to process information, learn from data, and make informed decisions. Classification and regression are two fundamental tasks in supervised ML tailored to different types of data and problem domains. Classification algorithms aim to predict discrete categories for given inputs with different techniques including logistic regression, decision trees, random forests, support vector machines (SVM), l-Nearest Neighbors (k-NN) AND Neural Networks. Regression algorithms, on the other hand predict continuous numerical values. Decision trees and Random Forests (RF) can also be applied to regression tasks to get continuous outputs. Gradient boosting methods like XG Boost helps to product strong predictive model for weak learners that are effective in both regression and classification tasks [2]. Through the application of both regression and categorical algorithms, ANNs achieve high performance on complex tasks, addressing various challenges in drug development and other fields. ANNs replicate the structure of biological neural networks by using interconnected nodes (artificial neurons) that process input data, identify patterns, and generate outputs. This ability to model and understand complex relationships in data makes ANNs particularly effective for tasks requiring high accuracy and adaptability. The learning process in ANNs involves adjusting the weights of connections between nodes to minimize prediction errors, enabling the system to improve its performance over time through training. The use of ANNs in drug development spans various applications, including predicting drug efficacy, optimizing formulation processes, and identifying potential adverse effects. By harnessing the power of deep learning, ANNs contribute to more efficient and effective drug development pipelines, ultimately enhancing the quality and safety of pharmaceutical products [3]. SVM, RF, XG boost were the most commonly used ANN to model complex, linear and non-linear functions of inputs [4, 5].

Dosage forms include tablets, capsules, liquids, creams, patches and suppositories each tailored for various routes of administration such as oral, topical, nasal and parenteral application based on the patient needs. Liquid dosage forms are pharmaceutical formulations that comprise one or more active pharmaceutical ingredients (APIs) along with a variety of excipients, such as solvents, sweeteners, colorants, preservatives, surfactants, emulsifying agents, stabilizers, etc. The development of these formulations is inherently complex, necessitating a profound understanding of the physicochemical properties of the compounds involved. Moreover, it requires a comprehensive grasp of pharmacokinetics and pharmacodynamics to ensure optimal therapeutic efficacy and safety. The intricate interplay between the API and excipients must be meticulously managed to achieve the desired solubility, stability, and bioavailability, ultimately leading to a product that meets stringent regulatory standards and delivers consistent therapeutic outcomes. Ensuring the quality and safety of pharmaceutical products is of utmost importance, necessitating strict adherence to rigorous regulatory requirements. Drug development of monophasic and biphasic liquid dosage form contains several processes including pre-formulation, formulation development and manufacturing. A large number of critical parameters must be considered during the formulation development process such as solubility, solvent selection, crystallinity, excipient compatibility, salt formation, stability, permeability, scale-up [6]. The viscosity of fluids significantly impacts manufacturing system performance by affecting friction losses during liquid flow. This effect is particularly pronounced in non-Newtonian fluids, which exhibit shear-thinning and shear-thickening behaviours. The utilization of non-Newtonian fluids has notable repercussions on centrifugal pump performance, impeller function, and is associated with various operational malfunctions [7].

The complexity of manufacturing processes escalates and the demand for operational efficiency intensifies, the integration of AI has emerged as a transformative approach. AI holds the potential for processing and analysing vast amounts of multivariate data, speeding up the manufacturing process, establishing a link between formulation characteristics and processing variables, and understanding a solution for challenging formulation development issues. This integration improves the productivity, product quality, detecting faults, anomalies, cost savings, optimizing production work flows and overall edge over the competitors. A seamless network of connected machinery and sensors will be produced by combination of AI with internet of things (IOT) devices. AI would also monitor equipment wear and tear, guarantee constant product quality by analyzing real-time data from these devices. By automating record keeping, auditing and documentation procedures, AI will simplify regulatory compliance for manufacturing of liquid dosage form. Thus, guaranteeing the production facilities for pharmaceuticals continue to adhere to the changing requirements of the industry. The application of ANN helps to overcome the intricacies in the process better, especially in simultaneous optimization process. The system employs learning modalities for pattern recognition, utilizing the classification of multidimensional dataset inputs to generate predictions and analytical suggestions for output assessment [8]. Modeling and simulation in research and development was recognized by US Food and Drug Administration (USFDA) with the context of digital pharmaceutical development [9]. USFDA has developed comprehensive policies to provide clear guidance for the utilization of AI in the development and manufacturing of drug products. These policies aim to establish a framework that ensures the safe and effective integration of AI technologies into drug product life cycles, encompassing areas such as product design, development and manufacturing processes. By delineating regulatory expectations and fostering innovation, these guidelines enhance the quality, efficacy and reliability of drug products, ultimately benefiting public health and advancing the healthcare industry [10]. Pharmaceutical development encompasses a science and risk-based systematic approach through Quality by Design (QbD), involving defined control ranges for individual Critical Material Attributes (CMAs) and Critical Process Parameters (CPPs) to ensure targeted product quality. In routine continuous manufacturing process, the integration of Process Analytical Technology (PAT) tools with AI facilitates the automatic analysis of diverse data types, enabling real-time decision-making [11]. This synergy enhances the efficiency and responsiveness of manufacturing operations by allowing for the continuous monitoring and control of critical process parameters. The AI-driven analysis of data collected through PAT tools ensures timely identification of deviations and optimizes process adjustments, thereby improving product quality and consistency while reducing the likelihood of production disruptions. This integration supports process engineering, scale-up, flexible design space, and the use of control charts to track process deviations and facilitate post-approval continual improvements. Furthermore, it enables the instant processing of regression model equations to systematically and rapidly evaluate all potential factors, ensuring a comprehensive understanding for quality product development [9].

**Fig. 2:**
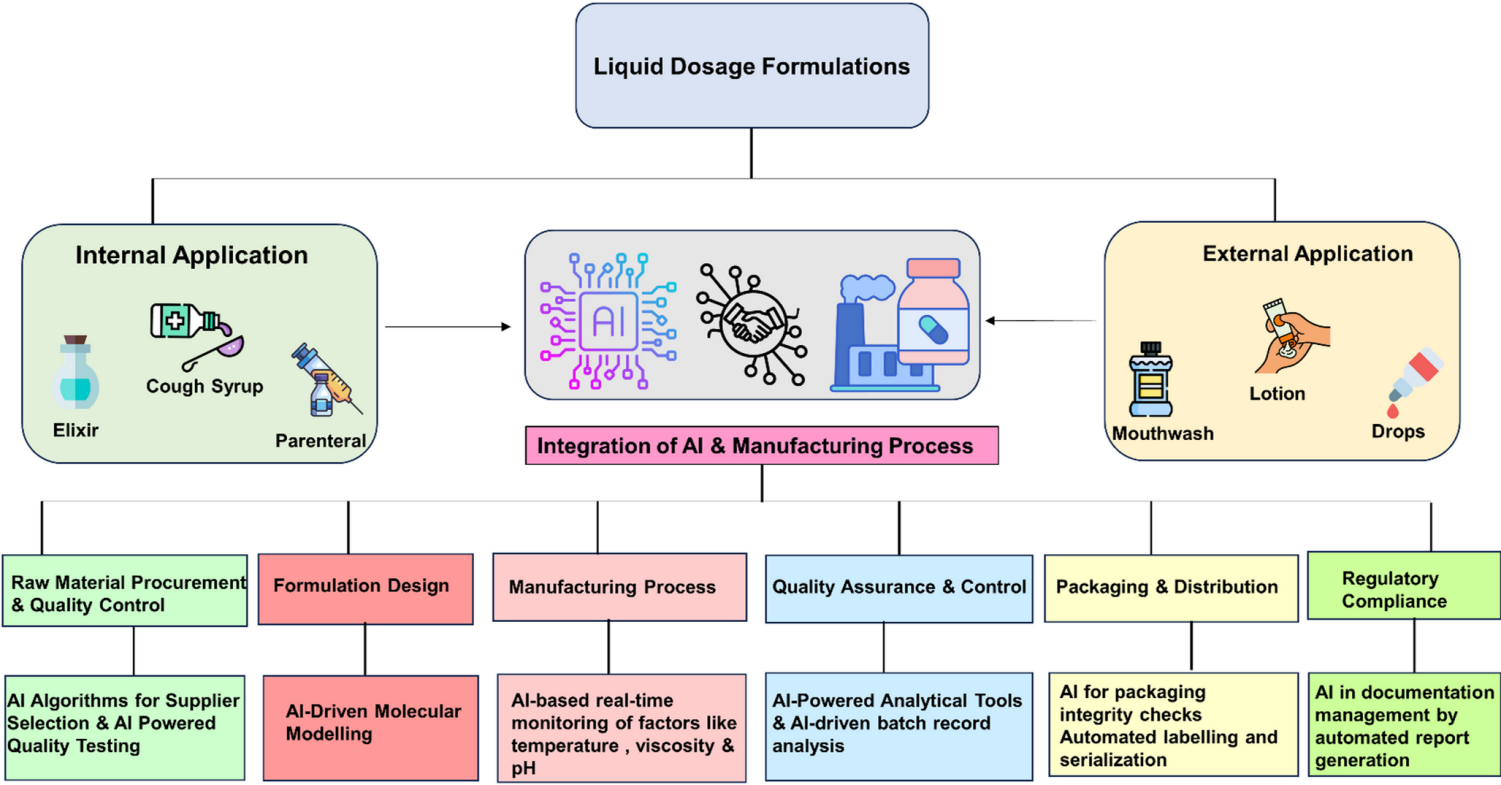
Schematic diagram of AI-assisted processes in manufacturing various liquid dosage formulations

Industry 5.0 is the next phase of industrial revolution that would leverage human-machine interactions and transform the manufacturing industries with new concepts of Augmented Intelligence (AuI) and cobots [12]. AuI represents the integration of AI with human intelligence, aimed at enhancing both machine capabilities and human potential. This symbiotic approach leverages the strengths of AI, such as data processing and pattern recognition, along with human cognitive abilities to create more effective decision-making processes and operational efficiencies. Robotic process automation has sparked innovations in industries, yielding substantial benefits for the global ecosystem characterized by increased control, reduced errors, drives sustained improvements and manages the resources. Collaborative robots or cobots are specifically designed to interact safely and effectively with humans in the industry. Unlike traditional industrial robots, cobots possess advanced sensing technologies and sophisticated algorithms that enable them to avoid collisions and work alongside humans to perform tasks with precision and adaptability in dynamic environments. The integration of AuI and deployment of cobots are key drivers in the transition from Pharma 4.0 to Pharma 5.0 paradigms [13]. This improves the process effectiveness and quickens the pace and precision of automation by interlinking machinery, processes, and systems to optimize performance in the industry. It enables manufacturers to adopt sustainable manufacturing practices, improve productivity, enhance product quality, and optimize workflow within the industry. In the context of industrial operations, the adaptation of AI facilitates a significant evolution in maintenance practices, transitioning from traditional reactive and preventive maintenance strategies to a more sophisticated predictive maintenance approach. Predictive maintenance leverages data analytics, ML and real-time monitoring to anticipate equipment failures before they occur, thereby optimizing maintenance schedules, minimizing unplanned downtime and executing the lifespan of assets. This not only enhances operational efficiency but also leads to substantial cost savings and improved reliability of industrial systems. However, the present work explores the real-time application of AI in liquid dosage form manufacturing processes as a means of preparing for smart manufacturing. Figure 2 illustrates AI-assisted processes in manufacturing various liquid dosage formulations. ML have already found several applications in drug discovery [14, 15] and solid dosage forms [16]. To the authors’ knowledge, the manufacturing processes for liquid dosage forms have not been thoroughly examined in the existing literature. Consequently, this paper aims to investigate real-time tools and techniques for process monitoring and modification in the most common manufacturing steps of liquid dosage forms.

## Liquid Dosage form in Pharmaceutical Applications

Liquid dosage forms are pourable pharmaceutical formulations consisting of one or more APIs and excipients dissolved or suspended in an appropriate solvent or solvent blend. These formulations are specifically intended for populations requiring rapid therapeutic action or those with difficulty swallowing solid dosage forms. Their ease of use, patient compliance, and adaptability to a broad demographic have contributed to their growing market demand in recent decades. For instance, acetaminophen-based liquid products such as syrups and suspensions exhibit high compound annual growth rates (CAGR) [17]. These are available as ready-to-use liquids or as powders for reconstitution and can be administered through various routes including oral, parenteral, nasal, ophthalmic, otic, inhalation, and topical. While oral formulations are nonsterile, parenteral forms are subject to sterility requirements based on clinical need and administration route [18].

### Monophasic Liquid Dosage Forms

Monophasic liquids consist of a single-phase system in which the API and excipients are completely solubilized in a suitable solvent. These systems are absorbed rapidly and efficiently. Monophasic oral liquids include mixtures, elixirs, syrups, draughts, and linctuses. External formulations include lotions, liniments, and collodions. Additional examples encompass applications such as mouthwashes, eye and ear drops, nasal sprays, enemas, and inhalations. Parenteral monophasic solutions are used as injections [19]. Products such as Amoxicillin oral solution, Benadryl syrup, and Phenobarbital elixir demonstrate the clinical utility of such systems, offering both therapeutic efficacy and improved patient compliance [20]. The formulation of monophasic liquids is guided by the Quality Target Product Profile (QTPP), which encompasses factors such as dosage strength, route of administration, and pharmacokinetics. Key attributes include solubility, stability, compatibility of drug and solvent, organoleptic properties, and appropriate packaging. Critical material parameters include pH adjustment, sterilization techniques, and concentration of preservatives. Critical process parameters involve maintaining homogeneity through effective mixing, agitation control, and filtration methods [21]. For parenteral products, both Small Volume Parenterals (SVPs) and Large Volume Parenterals (LVPs) require strict process control to ensure safety, efficacy, and stability [22].

### Biphasic Liquid Dosage Forms

Biphasic systems consist of two immiscible phases and include suspensions and emulsions. Suspensions are heterogeneous systems where the API is dispersed as insoluble particles in a liquid medium using suspending agents. In contrast, emulsions consist of two immiscible liquids, typically oil and water, stabilized with emulsifying agents. These are further classified into oil-in-water (O/W) or water-in-oil (W/O) emulsions based on the dispersed and continuous phases. Such formulations are employed to improve solubility and stability of poorly soluble drugs [23]. For example, Pepto Bismol (bismuth subsalicylate suspension) is used for gastrointestinal disorders, and Diprivan (propofol emulsion) facilitates intravenous anesthesia. Critical quality parameters for suspensions include particle size distribution, while for emulsions, droplet size uniformity is essential. The development process requires control of mixing, homogenization, and emulsification to maintain uniformity and avoid degradation or phase separation. Sterile conditions and appropriate packaging are essential to ensure product stability and safety [24].

Formulating both monophasic and biphasic systems requires a comprehensive understanding of the API’s physicochemical properties and their interactions with excipients. Excipients serve multiple roles, such as solubilizers, viscosity modifiers, preservatives, and stabilizers. Their performance depends on concentration and interaction with the API and other formulation components [25]. Factors such as morphology, particle size, surface chemistry, and wettability of the API and excipients must be characterized to ensure effective drug delivery and long-term stability [26]. Typically, manufacturing involves dissolving the API and excipients in solvents, suspending the API in a liquid medium, or dispersing in an oil/water phase. These processes occur in large mixing vessels equipped with thermostatic controls and mechanical stirrers. Component addition is standardized to ensure consistency across batches. As with all pharmaceutical formulations, liquid dosage forms must comply with stringent specifications to maintain batch uniformity and shelf stability. Rheological properties significantly influence liquid formulation behavior during manufacturing and administration. Increased viscosity can impede flow and pumping processes, necessitating proper evaluation during development [27, 28]. Rheological assessment informs decisions about pourability, syringability, and flow characteristics. Fluids are categorized as Newtonian or non-Newtonian based on their shear stress versus shear rate relationship. Many pharmaceutical liquids, such as emulsions and suspensions, exhibit non-Newtonian behavior like shear-thinning or shear-thickening [29]. These properties depend on the formulation’s previous shear history and the duration of applied force, which can manifest as thixotropy or rheopexy. These behaviors are crucial in optimizing process parameters such as mixing, filling, and stability of final products [30]. Suspensions present additional challenges due to their particulate nature. Parameters like particle size, zeta potential, morphology, and volume fraction impact flow behavior. Brownian motion and shear forces influence the movement of particles, which can alter the formulation’s viscosity [30]. In emulsions, rheology influences mixing, filling rates, and even product appearance. Droplet interactions, influenced by aeration, temperature, and additives, determine flow properties and visual attributes such as texture and creaminess. These considerations are especially critical for injectable formulations, where viscosity affects syringeability and injection force [31].

Process monitoring tools such as computational fluid dynamics (CFD) help simulate fluid flow and stress responses in reactors. Real-time monitoring of variables like heat flux, entropy, and internal energy is crucial during formulation and scale-up [32]. Primary packaging components, including ampoules, vials, and bottles, are designed to protect the formulation from external contamination and ensure proper delivery [33]. Stability studies are conducted to assess the interaction of packaging materials with the formulation [34].

Traditional large-batch manufacturing offers limited flexibility and struggles to meet evolving regulatory and market demands. The integration of AI under the Pharma 5.0 framework is transforming liquid dosage form manufacturing. AI enables predictive analytics, real-time monitoring, and automation, leading to greater efficiency, consistency, and compliance [35]. AI technologies assist in formulation optimization, anomaly detection, and minimizing manual intervention in hazardous tasks. Smart manufacturing integrates process design, development, and quality assurance within a single digital framework. This shift promotes innovation and accelerates time-to-market for liquid pharmaceutical products.

### Pre-Formulation Challenges for Mono and Biphasic Formulation

Monophasic liquid formulations face challenges in solvent selection, as the API must remain soluble and stable under varying pH, temperature, and excipient interactions. Poor solubility can lead to precipitation, while chemical instability (e.g., hydrolysis, oxidation) may degrade the API. Excipient compatibility is critical-preservatives, buffers, or co-solvents can induce degradation or alter bioavailability. Additionally, hygroscopicity may affect long-term stability, requiring moisture-resistant packaging. Biphasic liquid formulations introduce complexities like droplet/particle size control, which impacts stability and drug release. Sedimentation or creaming can occur if viscosity or interfacial tension is improperly balanced. Phase separation risks are heightened by temperature fluctuations or shear stress during manufacturing. Excipients (e.g., emulsifiers, thickeners) must be optimized to prevent coalescence or Ostwald ripening. These challenges necessitate robust pre-formulation studies, and often, the approach of advanced formulation approaches to optimize performance, stability, and manufacturability [36].

## Application of AI in Liquid Dosage Forms

**Fig. 3:**
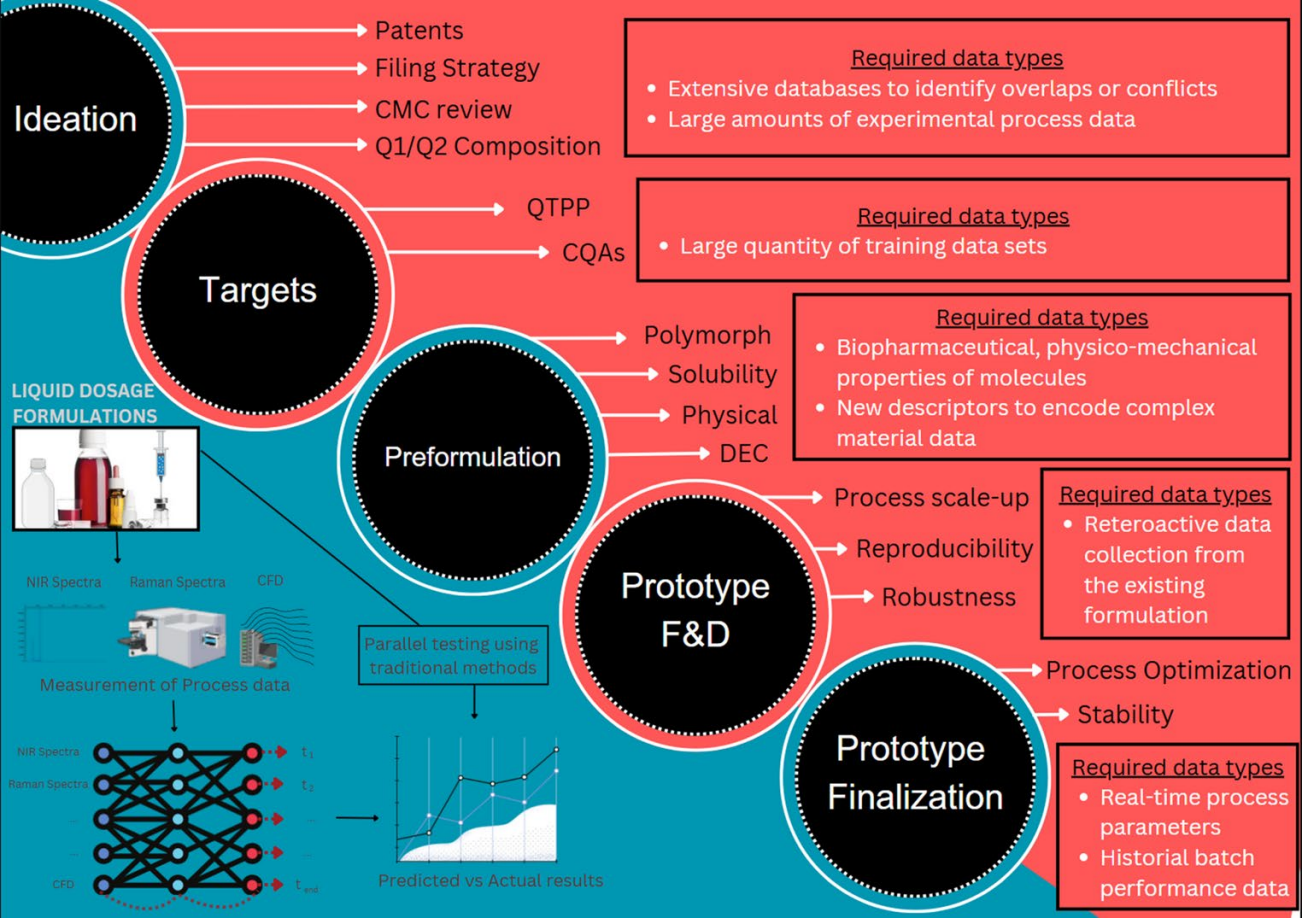
Integrating ANN in liquid dosage form manufacturing: A data-centric approach

The development of liquid dosage formulations, form initial conception to process scale-up, follows a structured framework. The ideation phase primarily focuses on defining the target market while considering regulatory requirements, patent landscape, and filing strategies. Once the formulation objectives are established, scientists define the Critical Quality Attributes (CQAs) to ensure product efficiency and compliance. Pre-formulation studies play a crucial role in formulation design by assessing the physicochemical properties API, including solubility, polymorphic characteristics and others, to optimize stability and bioavailability. Prototype formulation and development traditionally rely on trial-and-error methodologies to enhance robustness such as mixing, filtration, and homogenization are essential to ensure consistency in large-scale production. The incorporation of data from previous formulations facilitates the refinement of scale-up strategies, contributing to process efficiency and reliability. Finally, prototype finalization ensures process stability through comprehensive batch performance evaluations and real-time monitoring [37]. Conventional methodologies emphasize formulation optimization prior to large-scale manufacturing, relying on experimental investigations and retrospective analysis to achieve product quality and regulatory compliance. ANN enhances formulation process by accelerating data analysis as shown in Fig. 3, optimizing parameters, and reducing trail-and-error experimentation. AI-driven models utilize extensive datasets to predict formulation outcomes, improving reproducibility and robustness. This automation streamlines scale-up, ensuring efficiency, cost reduction, and enhanced pharmaceutical manufacturing precision.

The integration of advanced technologies such as blockchain and ML into the pharmaceutical sector, often referred to as the “smart industry,” has the potential to significantly enhance operational efficiency, transparency, and security. Blockchain technology, in particular, plays a critical role in combating counterfeit drugs, which pose severe risks to patient safety and result in substantial revenue losses for pharmaceutical companies [38]. Moreover, ML can be leveraged for data analysis, review processing, and providing personalized recommendations, thereby optimizing manufacturing processes and improving the overall customer experience within the pharmaceutical industry. Table 1 provides a comparative overview of AI-integrated software solutions tailored for formulation, process modeling, and quality optimization in the pharmaceutical production of liquid dosage forms, emphasizing the practical applications of these advanced technologies. ANN exhibit unique characteristics such as adaptability, nonlinearity and arbitrary function mapping capability, making them particularly suitable for forecasting tasks, where they generally deliver satisfactory performance [39]. Numerous studies indicate that deep learning frequently outperforms other machine learning techniques in variable prediction tasks. Extensive research has been conducted in this domain related to the manufacture of liquid dosage forms to advance the principles of smart manufacturing.

**Table 1:** AI-Driven software tools for liquid dosage form development and optimization

S. No.	Software Name	Key Features	Integration of AI	Application in Liquid Dosage Forms
1	QbDWorks	Quality-by-Design (QbD) framework for pharmaceutical formulation and process development	Applies ML to risk analysis, process optimization, and predictive modeling.	Stability, viscosity, and dissolution profiles prediction analysis [40]
2	SimSci PRO/II	Process simulation software for chemical and pharmaceutical manufacturing	Algorithms supported by AI for real-time optimization and modifications	Simulating the heating, chilling, and mixing procedures in the production of liquid formulation [41]
3	ZoomLab	AI platform designed for pharmaceutical applications	AI for formulation screening and predictive modeling	Assists in determining the best excipient combinations and interactions between drugs and solvents [42]
4	Aspen Plus	Process modeling and optimization software	AI-driven simulation for scale-up and efficiency	Helps to optimize ingredient quantities and scale-up liquid formulation proceses [43]
5	SuperPro Designer	Process modeling and cost estimation for pharmaceutical production	AI to evaluate alternative manufacturing routes and optimize resource allocation	Used for batch design and scale-up of liquid dosages [44]
6	PAT Solutions	PAT tools integrated with AI	Real-time quality monitoring and predictive maintenance	Ensures CQA during liquid dosage production [45]
7	LabDCT	Digital twin platform for laboratory and process testing	Utilizes AI-powered modeling to mimic production environments	Allows testing of formulations in virtual environments [46]

### AI in Preformulation

The integration of AI into pre-formulation studies provides a transformative approach to resolving the complex challenges encountered in both monophasic and biphasic liquid dosage forms as outlined in Table 2. In monophasic formulations, selecting an appropriate solvent that maintains the solubility and chemical stability of the API is crucial. AI tools such as ANNs facilitate the prediction of API solubility across varying solvent systems, reducing dependence on traditional trial-and-error experimentation. Advanced statistical models like Principal Component Analysis (PCA) and Partial Least Squares Discriminant Analysis (PLS-DA) have been employed to interpret spectral data from techniques such as Fourier-transform infrared spectroscopy (FTIR) and differential scanning calorimetry (DSC). These models support the rapid screening of excipients, aiding in the identification of potentially reactive agents and improving formulation robustness. Furthermore, multivariate models assess the hygroscopicity and moisture sorption characteristics of formulation components, enabling precise packaging decisions to safeguard long-term product stability. In biphasic formulations, controlling droplet and particle size is essential for preventing instability phenomena such as sedimentation, creaming, or phase separation. CFD and machine ML models are increasingly utilized to simulate homogenization conditions - such as shear rate and stress - to achieve desirable emulsion characteristics. Additionally, ANN-based models can correlate rheological data with sedimentation behavior in suspensions, predicting physical stability more accurately. AI also aids in optimizing emulsifier and thickener concentrations to prevent coalescence, Ostwald ripening, and viscosity-induced settling.

**Table 2:** Overview of formulation challenges and AI/ML-Driven solution in mono and biphasic formulations

S. No.	Formulation system	Key Challenges	AI/ML solutions	References
1	Monophasic systems	Solvent selection for API solubility and stability	ANN for solubility prediction based on physicochemical properties	[47]
2		API excipient compatibility (E.g., hydrolysis, oxidation, polymorphism)	PCA and PLS-DA for rapid screening of compatibility using FTIR and DSC	[48]
3		Hygroscopicity and moisture sorption affecting stability	Multivariate analysis to classify hygroscopicity and predict moisture uptake	[49]
4		Polymorphism impacting bioavailability and manufacturability	DSC and XRD coupled with PCA to identify stable polymorphs	[50]
5	Biphasic Systems	Droplet size control in emulsions (E.g., instability, coalescence)	CFD for shear stress optimization during homogenization	[51]
6		Sedimentation and phase separation	ML models to predict stability based on viscosity and interfacial tension	[52]
7		Uniform distribution of API in suspensions	ANN to optimize particle size distribution and suspension rheology	[53]

APIs are typically formulated into dosage forms to achieve the appropriate and consistent delivery volume. Pharmaceutical excipients include vehicle/ solvent, co-solvents, surfactants, preservatives, viscosity modifiers, emulsifying agents, flavouring agents, antioxidants, chelating agents, pH modifier, solubilizers, lyophilization reagents, osmotic pressure regulators, vaccine adjuvants etc., It is necessary to identify, quantify and predict potential interactions on the manufacturability, quality and performance of the excipients with the API. FormulationAI and PharmDE are innovative platforms designed to support pharmaceutical formulation development, particularly for liquid dosage forms. Wang et al. designed PharmDE, a knowledge driven expert system, was able to assess the risk of drug- excipient incompatibility [54]. This comprehensive database encompasses 532 data points derived from 228 meticulously selected papers. Based on this information and formulation research experiences, sixty guidelines for drug-excipient interactions were formulated. It features basic database searches, similarity-based data matching, drug incompatibility risk evaluations, and formulation incompatibility risk evaluations by integrating database searches with rule-based risk predictions. PharmDE employs logical reasoning for drug-excipient incompatibility risk assessment using the open-source cheminformatics software RDKit and the Python programming language. PharmDE is expected to accelerate drug formulation development. To facilitate easy access, a user-friendly website (https://pharmde.computpharm.org/) has been created allowing users to obtain compatibility information and conduct an incompatibility risk assessment. FormulationAI leverages advanced ML models to predict key formulation parameters such as solubility and stability across various drug delivery systems. It offers a user-friendly interface, making it suitable for real-time formulation design [55]. PharmDE, on the other hand, is a rule-based system that evaluates DE compatibility using mechanistic rues and literature data. While PharmDE ensures early-stage risk assessment, FormulationAI aids in optimizing formulations. Together, these tools can streamline liquid dosage form development, though limitations remain in data coverage, transparency, and regulatory integration. Hamid and Gupta utilized DL tools for excipient-excipient incompatibility prediction which conducts standard database searches for incompatibility, perform data matching via similarity searches, and assess risks associated with medication and formulation incompatibilities [56]. DL algorithms, employed for predicting excipient-excipient incompatibilities, have demonstrated superior accuracy is evaluated using random forest methods, which effectively identify the structural components responsible for the primary incompatibilities and key properties in pharmaceutical research and development. To assign probabilities to excipients, the system employs a confusion matrix: values exceeding 0.5 are considered inactive (lacking the incompatibility or property), whereas values below 0.5 are deemed active (possessing the incompatibility or property). The DE-INTERACT model (https://decompatibility.streamlit.app/) exhibited remarkable accuracy in rapidly predicting instances of drug-excipient incompatibility [57]. This DE-INTERACT was employed as reference standard in a recent study that utilized several ML techniques to develop an effective model for predicting drug-excipient compatibility [58]. Traditional methods such as DSC, FTIR, and chromatographic techniques are effective but can be resource-intensive. To streamline this process, *DE-Interact*, a machine-learning-based predictive tool designed to detect potential incompatibilities using PubChem molecular fingerprints and ANNs. The model was trained on over 3,500 drug-excipient combinations, utilizing 881-bit PubChem fingerprints to represent each compound. These fingerprints were concatenated to form a comprehensive 1762-bit input feature set, with outputs categorized as either “compatible” or “incompatible.” DE-Interact was validated using multiple ANN models optimized for accuracy, loss, and precision, achieving up to 99.6% validation precision. A noteworthy validation of the DE-Interact tool was its prediction of an incompatibility between paracetamol and vanillin. This prediction was confirmed through a series of analytical tests. DSC analysis showed a significant shift and broadening of paracetamol’s melting peak in the mixture, indicating thermal instability. FTIR spectra of aged mixtures revealed the disappearance of characteristic amide NH peaks, suggesting chemical interaction. HPTLC and HPLC further confirmed degradation or transformation of paracetamol in the presence of vanillin over time. These findings not only validated the DE-Interact model but also underscored its practical utility in early-stage formulation screening, where it can help prevent costly downstream failures. The staking model introduced in the study demonstrated superior performance compared to the DE-INTERACT model in detecting drug-excipient incompatibility. Specifically, the staking model successfully identified incompatibility in 10 out of 12 tested cases, whereas the DE-INTERACT model only detected incompatibility in 3 out of 12 cases during validation experiments. Recent studies validate the efficiency of AI models in predicting DE interactions and physicochemical properties. As shown in Table 3, tools like DE-Interact (ANN) achieve up to 99.6% validation precision, outperforming traditional methods such as DSC or FTIR in identifying incompatibilities. Similarly, ANN based solubility prediction models demonstrate robust alignment with experimental data, reducing reliance on trial-and-error approaches.

**Table 3:** Performance benchmarks of AI-driven models versus experimental methods in liquid dosage formulation

S. No.	Process	AI Model / Technique	Experimental / Traditional Method	AI Prediction Metrics	Experimental Metrics	References
1	DE Compatibility	DE Interact (ANN)	DSC, FTIR, HPTLC, HPLC	Validation precision: 99.6%	Confirmed incompatibility in 3/12 cases	[57]
2	DE Compatibility	Staking Model (ML)	DSC, FTIR, HPTLC, HPLC	Detected incompatibility in 10/12 cases	Confirmed incompatibility in 3/12 cases	[58]
3	Solubility prediction	ANN (10 physicochemical descriptors)	Experimentallog S values	Robust predictive performance on test datasets	Experimental data used for training and validation	[59]
4	Crystallization optimization	MLP + Genetic Algorithms	Experimental Measurements	MAPE < 7.22% for predicted values	Aligned with experimental results	[60]
5	Emulsion stability	ANN (Surface concentration)	Dynamic Light Scattering	Strong agreement between observed and predicted particle sizes	Experimental data used for validation	[61]
6	Microemulsion structure prediction	ANN (DSC-based)	DSC curves	Prediction accuracy: 90%, RMSE: 0.54 °C	Experimental data used for training	[62]
7	Settling velocity in fluids	SVR (Polynomial Kernal)	Experimental observations	R²: 0.92, RMSE: 0.066, MSE: 0.0044, MAE: 0.044	Experimental data used for validation	[63]
8	P-HPLC Optimization	ANN + Levenberg-Marquardt algorithm	DoE-based experimental results	Minimal discrepancies between predicted and observed values (optimal topology: 3:10:4)	Experimental data used for validation	[64]
9	Lyophilization Optimization	Digital Twin (QbD framework)	Trial-and-error experiments	Up to 300% productivity gains, 60–75% cost savings	Experimental data used for validation	[65]
10	Emulsified Liquid membrane stability	ANN (Span 80 surfactant)	Experimental rupture rate measurement	*R* > 0.997, RMSE < 0.051	Optimal stability achieved at 0.07% rupture rate	[66]

The inherent solubility of drug-like compounds has been predicted using ANNs, which is useful in choosing the right solvent for liquid dosage forms. The model developed in a study [59], was trained on a dataset of 270 drug-like molecules with experimentally determined log S values. The ANN model was trained using 10 physio-chemical descriptors as input features, enabling it to learn the relationships between molecular properties and solubility. It has demonstrated robust predictive performance on the test datasets provided by the second solubility challenge (SC-2). This included a 100-compound low-variance tight set and 32 compound high-variance loose set, indicating its effectiveness in handling challenging datasets. Despite the inherent difficulties, the ANN model exhibited superior predictive capabilities, underscoring its potential for solvent selection in the formulation of liquid dosage forms. The ability to predict the pH stability of a drug component under varying conditions is also essential for expanding solvent selection options [67]. Air exposure significantly impacts pH and storage conditions, often leading to increased degradation. A study examines the application of ANNs to predict the stability of esomeprazole, a sensitive API, under different pH conditions [68]. The ANNs demonstrated superior predictive accuracy compared to traditional methods by effectively modeling complex interactions between variables such as pH and temperature. The research also involved careful monitoring of critical factors during the lyophilization process to ensure stability. An MLP network was developed to predict esomeprazole’s stability, showcasing strong predictive capabilities and facilitating the creation of a design space for a 36-month shelf life. Stability studies confirmed that pH and temperature significantly influence esomeprazole’s stability, with assay results remaining within specified limits under controlled conditions. The study concludes that maintaining a pH range of 10.4–10.6 is crucial for stability, and the MLP network proved effective in optimizing the lyophilization process.

Crystallization is a critical unit operation in the manufacturing of liquid dosage forms, particularly in solution and suspension as it significantly influences drug stability and bioavailability. It determines key crystal properties, including purity, morphology, polymorphism, size, and size distribution, all of which significantly influence the quality and efficacy of the final products [69]. The MLP network was specifically used in a study [60] to predict drug crystallinity that is applicable for liquid dosage forms by building models based on experimental data from the production process of pharmaceutical ingredients in the purest form. It is trained using historical data to predict the crystal density of the drug, establishing its effectiveness in capturing nonlinear relationships between the structural information of the crystal and main process parameters. This MLP model generated satisfactory results in predicting liquid crystalline behavior when compared to experimental measurements, with a mean absolute percentage error (MAPE) for predicted values less than 7.22%. when combined with Genetic Algorithms (GAs), the MLP model offered the best means of determining the operational parameters required to get notable enhancements in the drug’s crystallographic and physical characteristics. The performance and quality of the drug product can be improved by this integration. The advent of PAT sensors including Raman, in situ XRD, in situ laser backscattering, and in situ process image microscopy has significantly advanced the field of crystallization by enabling the precise measurement of critical parameters, such as liquid phase concentration, particle size, morphology, and crystal form of the solid phase, in real-time. This technological development of sensor technology in real-time monitoring allows for the detection of polymorph transformations in crystallization processes, aiding in the control and optimization of crystal forms [70]. Recent advances in computational modeling, particularly DL algorithm facilitate precise optimization of taste-masked paediatric formulations, addressing medication adherence challenges. The models analyze formulation component interactions to predict effective bitterness suppression strategies. High-throughput screening is enabled through integrated approaches, including electroencephalogram for taste intensity analysis, bioelectric tongues, and taste organoids on a chip. Advanced analytical techniques including fluorescence spectroscopy, surface plasmon resonance, and molecular docking further elucidate taste mechanisms. However, model efficiency relies on comprehensive datasets encompassing diverse taste profiles. While this computational paradigm represents a transformative shift in paediatric formulation development, clinical validation remains imperative for broader implementation [71]. Tacrolimus, an immunosuppressant with a narrow therapeutic index, requires precise dosing to avoid toxicity or rejection in paediatric renal transplant patients. Traditional methods often fall short due to interindividual variability. This study leveraged ML to develop a dose prediction algorithm using pharmacokinetic data from 21 paediatric patients (671 samples). Nine ML models were evaluated, with the Extra Trees Regressor (ETR) emerging as the most accurate, achieving an R² of 0.8 and meeting regulatory standards. Key features like AUC and time significantly influenced predictions. External validation confirmed the model’s robustness across diverse paediatric cohorts. The algorithm’s success highlights AI’s potential to enhance personalized dosing in liquid formulations, ensuring safety and efficacy. This approach can be adapted for other narrow-therapeutic-index drugs, advancing precision medicine in paediatrics [72].

Topical preparation containing chitosan and kappa carrageen showed how well the ANN model predicted drug release characteristics and skin penetration of diclofenac sodium. The accurate predictions by ANN with R^2^ value of 0.9994 underscored the significant influence of formulation parameters on diffusion mechanisms. The comparison between the simulated output data forms the ANN model and the experimental data demonstrated a high degree of alignment, indicating the reliability of the model in predicting permeation efficiency. This alignment emphasises the model’s potential to save time and resources in experimental tests for future studies. Therefore, the performance in predicting drug permeability in liquid dosage formulations was commendable, demonstrating its potential as a valuable tool for pharmaceutical formulations [60].

### Formulation Development

By integrating physical properties and operations into the generation process, generative intelligence (GI) in material science can be employed to modify target material data. This helps to predict the material performance and develop novel materials [73]. Rapid evolution of GI in material science, recent advances in physics-informed GI models have transformed the field. Models including neural operators, diffusion models now integrate physical laws directly into the generation process, enhancing predictive accuracy and enabling novel material discovery. Unni et al. highlights this shift, emphasizing the need for large-scale, standardized datasets and multimodal architectures to bridge gaps between traditional ML and domain-specific constraints [74]. Failure Mode and Effects Analysis (FMEA) is a systematic approach used to identify potential failure modes within processes and assess their potential impact on outcomes and product performance [75]. AI integration improves traditional risk assessment methods in FMEA. AI’s capacity to analyze historical failure data allows for more precise predictions of potential failure modes, while its ability to monitor system performance in real time enables dynamic updates to FMEA assessments. This integration leads to more informed decision-making, as AI-driven predictive analytics suggest preventative measures, facilitating proactive risk management. Furthermore, AI automates the data collection and analysis processes, streamlining risk assessments and allowing professionals to focus more on implementing solutions rather than merely identifying problems. In a case study, FMEA was applied to evaluate the reliability of subsystems within a man-machine system, identifying risks and recommending corrective actions to mitigate them. The incorporation of AI would further enhance this process by enabling continuous monitoring and improvement of system reliability [76]. ML algorithms can analyze large datasets to detect patterns of unfairness or bias that traditional FMEA methods may overlook. Natural Language Processing (NLP) technologies can evaluate user feedback, providing deeper insights into user experiences, which can then be integrated into FMEA-AI to enhance risk assessments. AI-driven simulation tools allow organizations to anticipate and prioritize risks by modeling potential failure scenarios [77]. GI facilitates the sematic modification or deconstruction of input samples descriptors, thereby enabling the accurate generation of target samples and the analysis of intrinsic relationships between characteristics. These advanced techniques enhance the ability to identify CMAs by effectively generating target samples from input descriptors or fitting the posterior distribution of the target samples [78]. The systemic and efficient execution of experiments is ensured by the DoE. This methodology rigorously analyses the data collected on the specific phenomenon under investigation, facilitating the formulation of robust conclusions for product innovation and enhancement. Numerous challenges associated with integrating DoE and ML techniques to enhance innovation in liquid dosage form product development [79]. However, ongoing research is dedicated to improving this integration. The application of DoE and ML has uncovered significant opportunities and insights in the field of product innovation. Particularly, ANNs have been employed for the analysis of DoE data, demonstrating notable effectiveness in regression tasks [80].

To accurately estimate solubility, Samuel Boobier et al., utilized various descriptors which are crucial for capturing solute-solvent and solute-solute interactions [81]. A total of 22 descriptors were selected, encompassing thermal and electrical energies, solvation energy, orbital interactions, dipole moment, charge distribution, molecular volume, solvent accessible surface area, molecular weight, and the number of atoms in the solute. The inclusion of the experimental melting point as a descriptor underscores the necessity of experimental values in specific scenarios. These descriptors are vital for developing interpretable predictive models for solubility in various solvents, thereby enhancing statistical robustness given the relatively small dataset size. This study aims to replicate the physiochemical relationships between molecular properties and solubility across different solvents to develop more precise predictive models. In the employed, comparative evaluation of eight ML techniques for solubility prediction, non-linear models consistently outperformed linear regression models. Notably, the quality of the data and descriptors was found to have a more significant impact on prediction accuracy than specific ML model employed. The models developed in the research exhibit high predictive accuracy for solubility, aligning closely with the anticipated noise levels in the training data. These models provide valuable insights into the factors influencing solubility and dissolution processes in oral liquid formulations. The advancement of AI in pharmaceutics, exemplified by platforms like Schrödinger, has greatly impacted formulation development. Early progress in computational tools has laid the foundation for more advanced modeling and simulation techniques, which have now become standard practices within the pharmaceutical industry. Schrödinger provides advanced tools that facilitate the development and optimization of drug formulations, a key component of computer-aided formulation design [82]. These tools enable the modeling and prediction of various formulation outcomes, which is essential for the creation of safe and effective pharmaceuticals. Schrödinger’s computational tools provide the ability to predict key formulation parameters such as solubility, encapsulation efficiency, and drug release profiles [83]. This capability can substantially reduce the time and financial resources required for experimental testing. By combining computational predictions with experimental validation, the accuracy and efficiency of the formulation process can be improved, leading to more optimized designs. The software also enables the simulation of complex chemical interactions on various scales, from molecular interactions to broader physiological effects, which is particularly valuable in the development of innovative liquid dosage forms. Additionally, computational approaches like those offered by Schrödinger can streamline regulatory approval by offering a deeper understanding of formulation performance and identifying potential challenges before clinical trials. Incorporating these methods allows researchers to better predict and refine drug properties, enhancing overall formulation outcomes. Polymorphism plays a crucial role in influencing drug solubility, bioavailability, and other key physicochemical characteristics. The manufacturing process, often subjected to stressors like hydrolysis, oxidation, and reduction, underscores the necessity of accurately predicting polymorphic forms. ML offers a robust approach to this challenge by utilizing large datasets to identify intricate patterns that may elude traditional methodologies. This enables real-time monitoring and adjustments throughout production, ensuring consistent product quality. By employing advanced data mining techniques, AI can predict the emergence of alternative polymorphic forms, aiding researchers in evaluating crystal structure stability and making informed decisions during the manufacturing process [84]. The Cambridge Structural Database (CSD) is a vital tool in training ML models, allowing for the development of customized scripts that support the identification of potential polymorphic forms [85]. Such capabilities are particularly advantageous in Industry 5.0, where rapid development cycles are essential. Additionally, a study [86] utilizing the Link-Prediction Method demonstrated the potential to improve predictive models by generating incorrect cocrystal sets, thereby enhancing the identification of rare drug-drug cocrystals and contributing to a more efficient manufacturing process.

AI-driven data models excel in evaluating the impact of microstructural features on the homogenization process, as they have the capability to analyse extensive datasets and identify patterns and relationships that traditional methods may overlook. Through reinforcement learning, AI can further optimize homogenization conditions by automatically adjusting variables such as mixing speed and duration, thereby achieving optimal results with minimal time and energy consumption [87]. Moreover, the integration of sensors and algorithms within AI technologies facilitates continuous monitoring and control of the homogenization process, ensuring consistent quality. These AI-driven approaches optimize processes in food and cosmetics industries by predicting emulsion stability and homogenization efficiency, where precise control over liquid properties is critical for enhancing efficiency, improving product quality, and reducing waste. ANNs are highly advantageous in homogenization applications owing to their ability to model complex interactions among variables [88]. This ability is essential for the precise prediction of effective properties in heterogeneous materials. The model’s generalization capability and the prevention of overfitting are ensured through the implementation of regularization techniques such as weight decay and dropout. Given the proficiency of ML, and specifically neural networks, in predicting homogenized properties, surrogate modeling emerges as a viable alternative to expensive simulations. Reinforcement learning further refines the homogenization process by dynamically adapting based on real-time data within PAT frameworks, while DL methodologies are utilized to simulate nonlinear stress-strain behaviors in composite materials. Rotor speed and flow rate are pivotal factors in high shear mixers (HSMs) utilized for the production of liquid dispersions. The structural attributes of the rotors, notably the number of teeth, are essential for optimizing mass transfer efficiency, particularly at elevated rotor speeds [89]. Measuring the rheological properties during the manufacture is a tedious process. Misinterpretation of these properties can result in several issues, including significant pressure drops during the homogenization process, cavitation leading to the unintended breakdown of larger particles or droplets into smaller ones, and alterations in viscosity. A study [90] utilized ANN technique with the self-adaptive differential evolution algorithm (SaDe) to develop an optimum model for each rheological property. This model effectively optimized the circulation process without the need for any supplementary methods. CFD allows to simulate complex fluid dynamics problems, predict the behavior of fluids and optimize engineering design without having to perform expensive and time-consuming experiments [91]. A study employed CFD to simulate flow fields in HSMs, leveraging advanced capabilities for accurate modeling [92]. Given the low volume fraction of the organic phase (1%), a single-phase simulation was utilized to simplify calculations while effectively predicting flow characteristics. The multiple reference frame method was applied to accurately model the transient motion between the rotor and stator, which is crucial for simulating the dynamics of rotating machinery. Turbulence effects were incorporated, and boundary conditions included velocity inlets and pressure outlets, with a no-slip wall condition applied to the solid surfaces. The results offer significant insights for the design and optimization of inline high shear mixers used in liquid-liquid systems. The particle settling velocity in a fluid is significantly influenced by the fluid’s rheological properties, particularly in non-Newtonian fluids. In industrial processes involving solid-liquid flows, the settling velocity is a key parameter, representing the steady free-fall velocity of a solid particle in a stationary liquid when gravitational and buoyant forces are balanced [93]. For fluids such as Bingham plastics, a specific yield stress must be overcome for flow to initiate, and this stress can influence critical factors like homogenization, mixing parameters, and particle settling dynamics [94]. Machine learning techniques can be employed to evaluate and optimize such solid-liquid interaction processes in real-time. In a study [63] comparing various machine learning algorithms, including SVR and Multi-Layer Perceptron MLP, the SVR model with a polynomial kernel demonstrated the highest performance, achieving an coefficient of determination (R²) value of 0.92, indicating a high degree of predictive accuracy for the modeling the settling velocity of a sphere in Newtonian and non-Newtonian fluid. The model was validated using a ten-fold cross-validation technique to ensure robustness and generalizability. Additionally, the performance metrics on an independent test dataset included a RMSE OF 0.066, Mean Squared Error (MSE) of 0.0044, and Mean Absolute Error (MAE) of 0.044. These values underscore the model’s strong predictive capability and low deviation from experimental results. A comprehensive feature analysis using leave-one-feature-out (LOFO) validation further confirmed the significant correlation between the selected input features and experimental observations, thus strengthening the model’s experimental reliability.

In the pharmaceutical industry, Air Handling Units (AHUs) play a crucial role in ensuring the maintenance of dust-free, clean air, while also providing the necessary heating and cooling functions to regulate supply air temperature and humidity levels. These measures are in strict adherence to FDA regulations and are aligned with current Good Manufacturing Practices (cGMP) [95]. The integration of AI with AHUs in HVAC systems significantly enhances monitoring and control by analysing sensor data in real-time, thereby ensuring optimal environmental conditions such as temperature and humidity—critical factors for adherence to US and EU pharmacopoeia standards. Additionally, AI enables predictive maintenance, which not only reduces costs but also minimizes downtime by anticipating equipment failures. This integration further optimizes energy efficiency, potentially recovering significant energy expenditures. Moreover, it ensures consistent environmental conditions and facilitates data-driven decision-making by processing extensive data from HVAC systems as part of quality control measures [96]. AI enabled predictive maintenance in HVAC systems further supports sustainability goals. Real-time monitoring and dynamic adjustments reduce energy consumption by 19–29%, as demonstrated in pharmaceutical cleanrooms. For a mid-sized facility, this equates to an estimated reduction of -15metric tons of Co_2_ emissions annually, while maintaining compliance with cGMP standards. Ultimately, this integration supports the development of smart buildings in line with industry 5.0 principles, leading to improved resource management and operational efficiency. A recent study [97] validates the effectiveness of y models in forecasting energy consumption within HVAC systems, with a particular focus on AHUs and absorption chillers. The study underscores the critical role of energy management in buildings, especially in air conditioning, which represents a substantial portion of energy usage. The research utilizes a Nonlinear Autoregressive with Exogenous Inputs (NARX) feedforward neural network model implemented in MATLAB, which has been validated for its accuracy in time series predictions, using operational data from an AHU and an absorption chiller. Key variables considered include ambient temperature, humidity, and seasonal factors. The ANN model was meticulously designed with specific structural and learning parameters, including data normalization and variations in training data size to enhance prediction accuracy. Performance was evaluated using the Coefficient of Variation of the (CV(RMSE)) and Mean Bias Error (MBE), in accordance with the guidelines set by the American Society of Heating, Refrigerating, and Air-Conditioning Engineers (ASHRAE) [98]. The results showed that the AHU model achieved a CV(RMSE) ranging from 19.42 to 19.53%, while the absorption chiller model demonstrated CV(RMSE) values between 27.12% and 29.39%, both of which fall within acceptable limits, thereby confirming the robustness of the ANN models for this application. Real-time monitoring of temperature and humidity is crucial in the pharmaceutical sector for ensuring the quality of sensitive products. Utilizing IoT-enabled sensors, the system continuously monitors key environmental factors, providing essential real-time data [99]. Additionally, the system tracks other variables such as light intensity and CO_2_ levels, to further ensure product quality. The integration of a data acquisition module facilitates the issuance of alerts when safe limits are exceeded, enabling immediate corrective actions. Compliance with regulatory standards is supported by a Real-Time Intelligent Monitoring and Notification System (RT-IMNS) mobile application, which offers visual representations of environmental trends and assists in remote monitoring. Moreover, a predictive model based on ANNs assesses the likelihood of product spoilage, thereby enhancing decision-making processes and ensuring the safety and efficacy of the products.

Furthermore, ML technologies have been utilized to enhance reverse phase high-performance liquid chromatography (RP-HPLC) techniques for the simultaneous testing of two drugs in emulsions [64]. It relies on critical output parameters including resolution, retention time, theoretical plate count (TPC), mean squared error, and correlation coefficient. This research utilized ANN coupled with the Levenberg-Marquardt (LM) algorithm to model the RP-HPLC process in a nonlinear manner. The minimal discrepancies between the predicted and observed values produced by the ANN linked with the LM model demonstrate the high accuracy and efficiency of the optimization process. The optimal network topology for the output parameters was determined to be 3:10:4, selected based on achieving the lowest MSE and the highest R^2^ values, thereby indicating the success of the optimization process. Henceforth, the DoE coupled ML is used as a reliable analytical method for the systematic optimization. Delivered dose uniformity represents a critical quality attribute in liquid dosage form, wherein the dose is quantified based on the number of drops or sprays administered as intended [100]. To ensure the uniformity of delivered dose (UDD), a stereo zoom microscope is employed to measure the orifice diameter in dosage form devices, particularly for eye and ear drops. High-resolution cameras capture real-time images of the orifice, and the image data is subsequently processed. The Motic Image Plus software facilitates precise calculations of the orifice diameter based on this data. Real-time data from the machine vision system can be integrated into a feedback control loop [101]. In cases where production complexities cause deviations between the measured and intended orifice diameters, prompt adjustments can be made to ensure that all manufactured dosage forms meet established quality standards. The performance of the stereo microscope can be monitored in real-time through the integration of ANNs with PAT. This capability enables rapid decision-making during image capture, ensuring the accuracy of calibration throughout the observation process. ANNs are particularly effective in identifying features and patterns in images, which assist in detecting changes in the orifice’s shape [102].

Based on its in vivo characteristics and in silico parameters, the GastroPlus Advanced Compartmental and Transit (ACAT) model serves as an essential tool for assessing oral exposure estimates, particularly for drugs classified under Biopharmaceutics Classification System (BCS) class I [103]. By integrating virtual simulations with experimental results, drug developmental approaches, such as population pharmacokinetics and physiologically based pharmacokinetic (PBPK) modeling accelerate the drug development process. These techniques significantly improve predictability and reliability in drug development. AI-based modeling software is employed to predict a drug’s pharmacokinetic behaviour and biological effects based on various principles [104–106]. In the context of liquid dosage forms, PBPK models are particularly valuable for predicting in-vivo plasma concentrations. These models incorporate the physicochemical properties of the drug, dissolution profiles, and absorption, distribution, metabolism, and excretion (ADME) data. By simulating the behavior of formulations within the body, PBPK models identify key factors influencing pharmacokinetics. This predictive capacity is essential for ensuring that liquid formulations, such as oral solutions, syrups, elixirs, suspensions or emulsions are designed to optimize drug absorption and therapeutic efficiency [107]. The application of PBPK modeling in the development of liquid dosage forms allows for a more detailed understanding of the interaction between formulation characteristics and physiological factors [108]. This, in turn, facilitates the optimization of formulation design and improves development efficiency. By more accurately reflecting the in-vivo performance of the drug, PBPK modeling not only streamlines the development process but also increases the likelihood of success in clinical trials [109].

Statistical Process Control (SPC) serves as a valuable tool for trend analysis, particularly in the pharmaceutical industry for process monitoring and improvement. The initial step in SPC involves understanding the behavior of the process. Control charts generated by Minitab, which visualize process variation over time, can effectively represent the data [110]. This allows users to identify trends and patterns, which are crucial for comprehending the overall process. Establishing control limits in SPC requires historical data, and Minitab enables users to define these limits, facilitating the detection of out-of-trend (OOT) results and determining whether the process remains under control. Maintaining process stability and preventing out-of-specification (OOS) results are contingent upon the accurate establishment of these limits [111]. Once control limits are set, Minitab can be utilized for continuous process monitoring, allowing for the identification of any deviations from expected performance. This enables timely interventions to prevent batches from failing to meet specifications. By employing this systematic approach, pharmaceutical processes in the manufacture of liquid dosage forms can maintain consistent quality and ensure regulatory compliance. ML has provided confidence that predictive stability modeling approaches, incorporating statistical tools, prior knowledge, and industry expertise, can offer robust and reliable predictions for the shelf-life of medicinal products [112]. These models enable the inclusion of comprehensive real-time stability data in regulatory submissions at the initial filing stage, thus facilitating the expedited availability of new drug products.

### AI Applications in Manufacture of Specific Dosage Forms

#### Solution

Solutions are extensively utilized in pharmaceuticals due to their high dosing flexibility and rapid onset of action. However, the manufacturing of such systems demands careful attention to various critical process parameters, including tank size, tank jacket temperature, impeller type, mixing speed and duration, homogenization method and duration, product temperature, feeding speed, filter size and partition coefficient, membrane filter capacity, filtration speed, solution viscosity, pump performance, and filling pump adjustments [113]. The integration of ANN for the optimization of these parameters significantly enhances overall performance of the dosage form manufacturing. Mixing time is influenced by multiple factors, such as system geometry, equipment configuration, fluid properties, and measurement techniques. In laminar flows, mixing times are significantly longer compared to turbulent flows, with non-Newtonian fluids exhibiting much longer mixing times relative to Newtonian fluids. The placement and type of impellers play a crucial role in enhancing mixing efficiency. Predictions of mixing time were generated using a MLP model and compared with experimental values, revealing varying levels of accuracy across different configuration. The analysis demonstrated that fluid flow characteristics, impeller positioning, and pumping direction had a considerable impact on the accuracy of mixing time predictions. This investigation also assessed mixing time by altering impeller eccentricity and testing different impeller types, including propellers and turbines [114]. The FDA recommends using high-speed photography or laser light sheets with high-speed digital cameras to accurately capture the spray’s development. Two orthogonal side views are preferred for comprehensive assessment, although one side view may suffice for in vitro equivalence testing. Automated image analysis is favoured over manual methods due to its reproducibility and reduction of operator bias. Key parameters such as plume angle, width, and height should be measured during the spray’s fully developed phase to ensure a thorough characterization. A study employs a Deep Neural Network (DNN) to predict spraying patterns using a dataset comprising 11,560 data points, each representing different variables [115]. The implementation of the DNN model effectively reduces the number of experimental trails required to investigate the influence of various parameters on plume geometry, thereby conserving both time and resources. This model provided high-accuracy predictions, enhancing the efficiency of the investigative process.

**Fig. 4:**
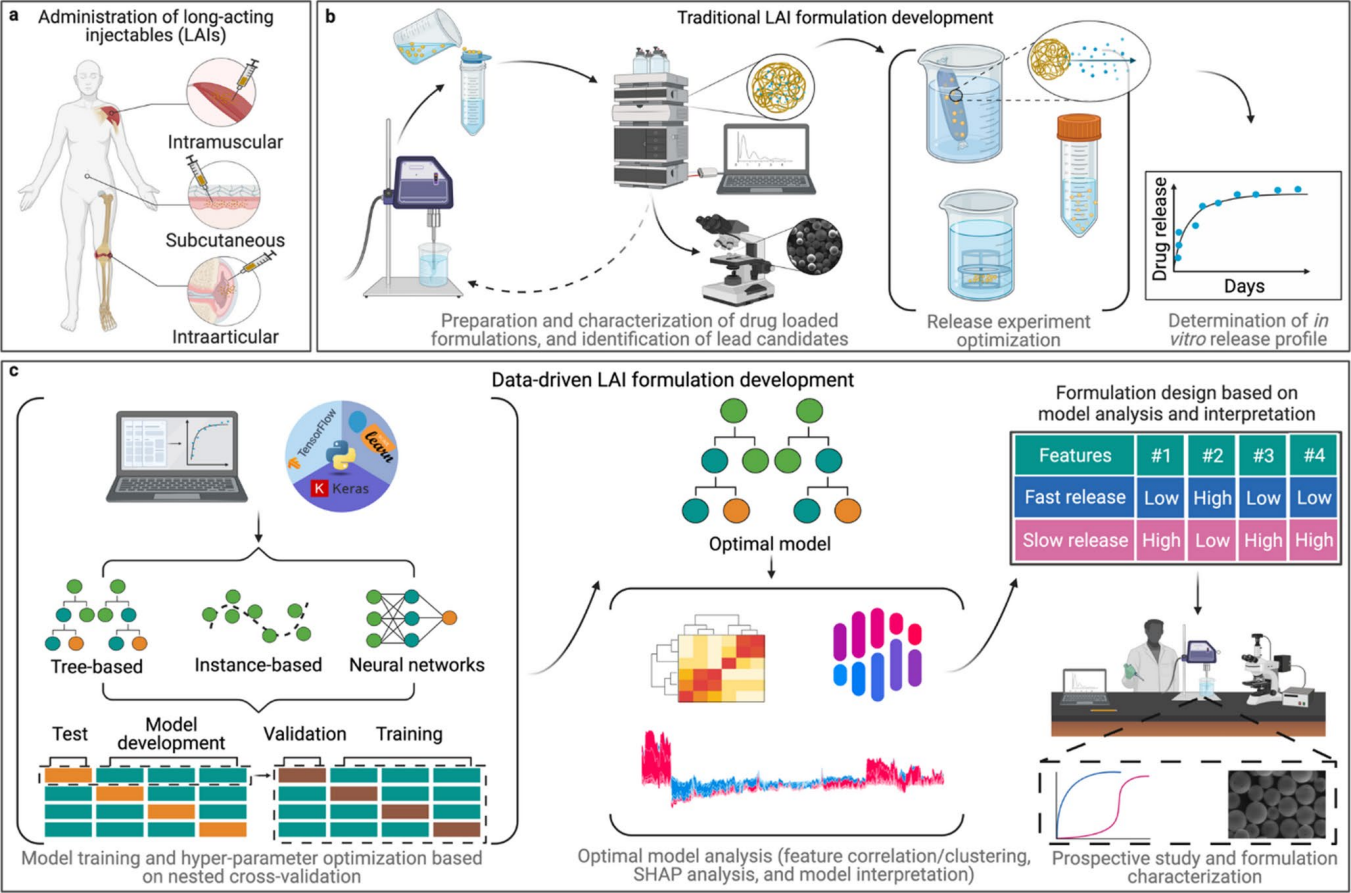
Schematic representation showing data-driven and conventional formulation development methods for long-acting injectables (LAIs), distributed under the usage, distribution, and reproduction are all allowed without restriction in any format as per the Creative Commons Attribution 4.0 International License. http://creativecommons.org/licenses/by/4.0/ (accessed on 16 Feb 2025) [118]

ML models enhance critical formulation attributes such as pH, osmolarity, solubility, stability, and viscosity by analyzing formulation ingredients and production processes [116]. These models also leverage process variables and historical data to identify potential contamination, stability issues, and regulatory deviations. During parenteral manufacturing, these monitoring systems enable real-time examination of key process parameters. For instance, particles adhering to, moving along, or sinking to the inner surface of the container can be analyzed to minimize non-compliance. By allowing liquid to flow inside the container, DL algorithms can track particle trajectories, while high-resolution imaging captures particle behavior [117]. It is particularly important to differentiate between bubble formation and particle formation, as bubble formation represents a critical issue contributing to parenteral batch failure. A study explored the key parameters influencing the design of Long-Acting Injectables (LAIs), specifically focusing on PLGA-based microparticles to achieve both sustained and rapid drug release profiles [118]. ML models were employed to train and predict drug release kinetics. The practical evaluation involved the release profiles of salicylic acid (fast release) and Olaparib (slow release), which aligned well with the model’s predictions, providing a robust basis for future technological advancements in the development of LAIs. The integration of DoE with QbD methodologies facilitated a comprehensive understanding of the process, allowing for the identification of potential CMAs and CPPs that could impact the quality of DepoFoam formulations [65]. This approach provided a systematic framework for experimental design, enabling the identification of key factors and ensuring the validation of outcomes through a structured and process control. Another study by Bannigan et al. [118]. developed LAI using ML models to forecast the release of experimental medications from intricate drug delivery devices. The data-driven and traditional formulation development approaches for LAI are depicted in Fig. 4. This technique is called as “data-driven LAI formulation development.” The models that were developed might be used as a basis for developing new LAIs. The study demonstrated that a data-driven approach might be used to develop medicinal formulations, potentially saving money and time. Lyophilization, or freeze-drying, is the preferred method for enhancing the long-term stability of small-molecule pharmaceutical products by converting an unstable molecular solution into a solid cake or powder through sublimation. Despite its advantages, lyophilization is a time- and energy-intensive process, and designing the cycle using a trial-and-error approach may result in elevated production costs [119]. Therefore, optimizing the process at every stage is essential to developing an efficient and reliable lyophilization cycle. The duration of freeze-drying cycle development is influenced by product complexity, team expertise, and resource availability, often taking several weeks to months. Traditional approaches require labour-intensive experiments including trial runs and iterative optimizations. Integrating QbD with a digital twin framework significantly accelerates this process by enabling predictive modeling grounded in scientific understanding. In a study, a digital twin was employed to optimize primary drying conditions, incorporating key parameters such as critical temperature (Tc), vial heat transfer coefficient (Kv), maximum sublimation flux (Jmax), and dry layer resistance (Rp). Controlled nucleation improved process reproducibility, while model simulations allowed for safe process design by maintaining product temperatures below Tc. The complete optimization using the digital twin required 14 working days and 540 mL of product solution. For subsequent formulations, development time reduced to five days. The validated pseudo-stationary model demonstrated strong agreement with experimental data, enabling faster, safer, and more cost-effective lyophilization with up to 300% productivity gains and 60–75% cost savings [120]. The integration of AI-driven twins in lyophilization not only accelerates process development but also significantly enhances sustainability. By optimizing primary drying conditions (e.g., critical temperature, vial heat transfer coefficient), AI reduces energy consumption by 60–75% compared to traditional trail-and-error approaches, translating to substantial cost savings and lower carbon footprint in large-scale production [117]. Maintaining consistent product quality during scale-up from laboratory to production requires a thorough understanding of equipment design and operational capabilities. Such knowledge mitigates the risk of failure when transitioning from laboratory to commercial-scale manufacturing by enabling the replication of temperature profiles across different production sites. This can be achieved through a hybrid approach that integrates CFD with machine learning techniques, specifically Adaptive Neuro-Fuzzy Inference System (ANFIS), SVR, and Poisson Regression (POR), optimized using the Cheetah Optimizer (CO) to improve temperature estimation [121].

Parenteral drug products must meet strict standards by being free from viable microorganisms, pyrogenic substances (specifically referring to minimal levels of bacterial endotoxins), and visible particulate matter [122]. While sterility tests cannot guarantee absolute sterility, a successful test indicates the absence of contaminants in the sample. The United States Pharmacopeia (USP) < 71 > outlines measures to prevent microbiological contamination during sterility testing, alongside appropriate sampling to monitor operational conditions [123]. Culture media, such as Soybean-Casein Digest Medium and Fluid Thioglycollate Medium, are employed to support the growth of fungi, aerobes, and anaerobes. Specialized procedures are necessary for the testing of pharmaceuticals, prefilled syringes, oily solutions, ointments, and sterile equipment, as these processes are constrained by the extended incubation periods required. A study by Dierks et al. [124] focuses on enhancing sterility testing, a critical component of current Good Manufacturing Practices (cGMP) for human health products, by introducing an automated approach using DL. This research employs convolutional autoencoders and CNNs to detect microbial growth, with sodium phosphate buffer and inoculated samples serving as test subjects. The system captured 46,070 top-view images of test vessels using Raspberry Pi Zero W computers, which were then processed to train models for microbial detection. The model achieved high classification accuracy, particularly for *C. sporogenes* and *P. aeruginosa*, though *S. aureus* presented challenges due to its morphological similarities with other organisms. The study highlights the potential of automating sterility testing, improving efficiency and accuracy, and lays the foundation for future advancements in real-time contamination detection and expanded microorganism applications. Clean rooms are categorized based on their capacity to regulate airborne particulate contamination, as specified by the US Federal Standard 209E [125]. Essential design considerations for clean rooms include the management of airflow, the efficacy of filtration systems, and the selection of construction materials, with AHUs playing a pivotal role in sustaining the required levels of cleanliness. Adherence to these standards is imperative for industries that depend on controlled environments, as it ensures both the maintenance of product quality and the protection of the health and safety of personnel. AHUs are vital for supplying pre-filtered air to cleanrooms in the manufacture of parenteral, which are essential for producing sterile products. The use of HEPA filters in conjugation with AHUs that is further coupled with AI support product quality, regulatory compliance, and operational efficiency [126]. The Programmable Logic Controller (PLC) utilizes an on-off control mechanism with hysteresis loop to achieve precise temperature regulation, while also incorporating humidity control to ensure thermal stability [127]. By calculating the necessary airflow, the system adjusts fan speeds to maintain optimal air quality and pressure conditions. This approach is aligned with the objectives of Industry 5.0, offering considerable potential for energy efficiency. Moreover, it serves as a promising framework for similar implementations in the pharmaceutical industry. Accurate control of pyrogens in parenteral dosage forms is of paramount importance, though existing detection methods involve multistep processes that are labour intensive, time-consuming, and unsustainable. A recent study introduces an aptamer-based biosensor for the real-time optical detection of endotoxins. The system integrates ML techniques with distance-dependent scattering of gold nanoparticles (AuNPs), EndoNet, and surface-enhanced Raman scattering (SERS) to significantly enhance both the accuracy and efficiency of endotoxin detection [128–130].

#### Suspension

The manufacture of suspension requires meticulous consideration of factors such as granulator type, impeller design and placement, product temperature, homogenization process, filter type, filter partition coefficient, filtration speed, and pump characteristics. These parameters critically influence key quality attributes, including dissolution profile, formulation density, viscosity, particle size and distribution, and sedimentation rate and speed [113]. ML algorithms, including Keras and LightGBM, were employed to predict nanosuspension particle size and polydispersity index (PDI) [131]. The study demonstrated strong predictive performance of the machine learning models, particularly for PDI in ball wet milling datasets and high-pressure homogenization methods. However, predictions for antisolvent precipitation (ASP) were less reliable, likely due to limitations in the available data. Optimal microfluidization settings depend on several variables, including pH, temperature, material properties, solvent type, number of passes, and processing pressure. Different materials require specific pressures, and increasing the number of passes through the microfluidizer can enhance its effects, depending on the substance being processed. Material characteristics, such as whether they are proteins or polysaccharides, also influence the ideal conditions. Temperature control is essential to prevent thermal degradation, while the choice of solvent affects the interaction between the material and the applied forces [132]. Additionally, certain properties, uch as protein water-holding capacity, can be improved by pH adjustments, such as using acidic conditions. Optimizing these parameters is crucial for maximizing the effectiveness of microfluidization in food applications. ML can significantly enhance high-throughput screening of microfluidization parameters by automating the experimental process and employing robotic systems to rapidly collect data on how various conditions impact suspensions [133]. This approach accelerates the optimization process while improving the reliability of the results. Integrating ML with mechanistic models enables the development of hybrid approaches that combine data-driven insights with established scientific principles. Such integration enhances the accuracy of predictions regarding particle behavior during micro fluidization, facilitating more effective process optimization. The operational efficiency of mechanically stirred tanks, particularly in suspensions, is dependent on a thorough understanding of the system’s hydrodynamics. To accurately represent the interactions between the solid and liquid phases, CFD models incorporate interphase forces, such as drag and turbulent dispersion. The complexity of the flow necessitates the use of both an interface model and a turbulence model to close the Reynolds-averaged momentum equations. Particle behavior is influenced by three primary forces: gravity, buoyancy, and drag, while particle characteristics and the effective viscosity of the slurry affect terminal velocity. By incorporating the average slurry density, CFD can reliably predict the power number in solid-liquid stirred tanks and reveal trends related to initial solid loading and viscosity [134]. Recent advancements in ML coupled rheology analysis have shown promising results in predicting and enhancing the stability of nano emulsions. By integrating rheological data with ML algorithms, researchers can uncover complex relationships between formulation parameters (e.g., surfactant concentration, droplet size) and stability outcomes. For instance, studies have employed ANNs and Gaussian process regression (GPR) to model the viscoelastic behavior of nano emulsions, enabling accurate predictions of shelf life and phase separation. Physics-informed ML approaches, such as rheology-informed neural networks (RhINNs), further enhance predictive capabilities by embedding physical laws into the models. This work highlights the success of these techniques in optimizing nano emulsion formulations, demonstrating improved stability and performance [135]. High-resolution imaging technology, combined with controlled ultrasonic agitation, enables rapid image acquisition and quantitative analysis of suspension stability [136]. This technique is not limited to pharmaceutical applications but has the potential to benefit other industries handling multiphase systems.

#### Emulsion

The inherent thermodynamic incompatibility between water and oil molecules in an emulsion leads to instability, ultimately causing the emulsion to break down over time. However, the use of micro- and nano-emulsions offers several advantages, including improved long-term stability, enhanced optical clarity, and increased bioavailability of the API [137]. The use of surfactants should be minimized to reduce potential toxic effects, while the concentration of poorly soluble drugs in the final formulation should be maximized by increasing the internal phase concentration [61]. These constraints inherently limit the options available for controlling particle size and viscosity. ANNs were employed to predict key emulsion properties, including particle size, zeta potential, conductance, and viscosity. The ANNs were primarily focused on predicting particle size, using formulation inputs such as surfactant and cosurfactant amounts, which significantly influenced the outcomes. Surfactant concentration, particularly, was identified as a dominant factor. The model was trained using dynamic light scattering (DLS) data, demonstrating strong agreement between observed and predicted particle sizes [138, 139]. ANNs were employed to predict the structures of microemulsions based on compositional data and DSC results. Two distinct ANNs were developed: one designed to forecast microemulsion types from compositional data, and the other utilizing DSC curves. The DSC-based model achieved a high prediction accuracy of 90%, while the composition-based ANN, with a simpler architecture, also effectively predicted microemulsion types. Both models were trained on a dataset of 170 microemulsion samples and demonstrated strong performance, with the DSC-based model producing a RMSE of 0.54 °C. Thus, ANN model reduces the experimental workload and enhancing the understanding of microemulsion structures [62]. To achieve an optimal nano emulsion formulation, it is crucial to maintain surfactant and co-surfactant concentrations within the recommended thresholds, while minimizing oil content to mitigate cytotoxic effects [140, 141]. The ANN model demonstrated that surfactant concentration is the primary factor influencing nano emulsion stability, whereas oil concentration plays a significant role in determining cytotoxicity [142]. AI has streamlined the design of microemulsions and created quantitative structure-property relationship (QSPR) models for predicting drug solubility in self-emulsifying drug delivery systems (SEDDS) [143]. Mechanical methods, including high-pressure homogenization and high-amplitude ultrasound, are increasingly integrated with ANNs to optimize the production of nano emulsions with droplet sizes below 500 nm. While high-pressure homogenization is effective, it necessitates a pre-prepared coarse dispersion, which is a notable limitation. In contrast, ultrasound techniques, leveraging acoustic cavitation to generate intense shear forces, present a more efficient alternative for nano-droplet formation. The application of ANNs further enhances this process by optimizing parameters, such as amplitude, to achieve optimal results with ultrasound [144, 145]. The ternary phase diagram serves as a critical tool for illustrating the phase behavior of three-component systems. These diagrams are instrumental in delineating the conditions for formation, identifying compositions that maximize emulsion stability, and defining regions of stability. The application of software tools, such as Pandat, significantly enhances the efficiency of high-throughput phase diagram calculations, facilitating the rapid analysis of phase equilibria data across various compositions and temperatures [146]. Additionally, Pandat aids in the organization of large datasets, which is essential for identifying phase boundaries and coexisting phases pertinent to material design. The incorporation of ML techniques, including support vector machines and random forests, further improves the accuracy of phase diagram predictions, thereby increasing the efficiency of exploring novel material systems and reducing the number of required experimental tests [147]. A study optimized the stability of an emulsified liquid membrane (ELM) using an artificial neural network (ANN). The ELM, composed of Span 80 surfactant, n-hexane diluent, and nitric acid internal phase, was tested for parameters like surfactant concentration, emulsification time, and stirring speed. Optimal stability (0.07% rupture rate) was achieved at 2% Span 80, 3 min emulsification, and 250 rpm stirring. An ANN model predicted internal phase leakage with high accuracy (*R* > 0.997, RMSE < 0.051). The ANN’s effectiveness was confirmed through residual analysis, demonstrating its potential for industrial ELM applications by enhancing stability and reducing experimental trials [66].

## Collaboration Between AI and Industry

**Fig. 5:**
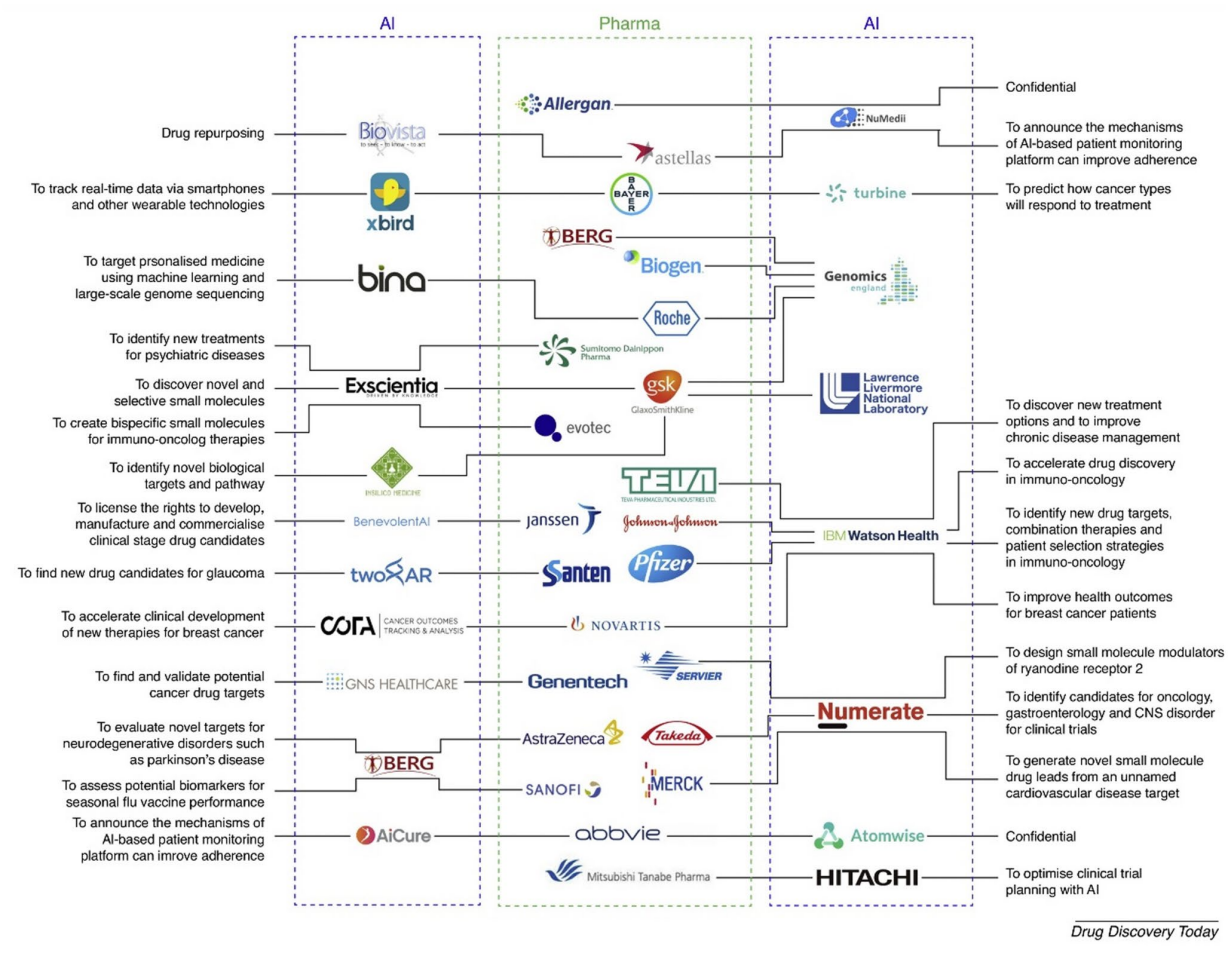
Schematic representation of collaborations between pharmaceutical corporations and artificial intelligence (AI) in terms of drug development. Reproduced with permission from Mak et al. [149]. copyright: © 2019, Elsevier, All rights reserved

Pharmaceutical companies that fully integrate AI use cases throughout their organizations have the potential to significantly enhance their operating profits, potentially doubling current figures, through increased revenues and decreased costs. The AI market in manufacturing is projected to reach USD 20.8 billion by 2028, increasing from USD 3.2 billion in 2023. This market is expected to grow at a CAGR of 45.6% from 2023 to 2028 [148]. The manufacturing sector is characterized by continuous evolution and the ongoing introduction of new technologies. Many manufacturing companies are actively exploring GAI applications on their production floors. As this technology advances, it is anticipated that significant use cases will emerge, particularly in the areas of quality control, predictive maintenance, and operational optimization. Contributing factors to the growth of AI within the manufacturing ecosystem include the increasing demand for more digitalized manufacturing processes. In terms of drug research, Fig. 5 symbolizes collaborations between pharmaceutical corporations and artificial intelligence (AI).

## Challenges and Future Prospectives

AI models face substantial challenges related to transparency, primarily due to their dependence on complex algorithms, which can impede regulatory approval processes. These models frequently struggle to provide clear explanations of their prediction mechanisms, complicating researchers’ ability to interpret and trust the results. Furthermore, limited data availability for modeling specific systems can lead to less accurate or biased predictions [150]. For instance, the integration of DOE with ML presents challenges, such as difficulties in seamlessly coupling stages, vague descriptions of applications, and issues with data splitting that affect the design structure and distort outcomes. Advanced validation techniques are crucial for ensuring the reliability of ML models based on DOE data. However, fractured design selection within DOE and restricted experimental conditions exacerbates the difficulty of this integration. The empirical nature of ML algorithms further complicates uncertainty quantification, necessitating methodological improvements to enhance the synergy between DOE and ML. In situations where data is incomplete or inaccurate, models are prone to generating imprecise predictions. The application of AI in liquid dosage form development also remains constrained due to the challenges in assembling large, balanced datasets. Published studies often exhibit a bias toward positive outcomes, with retrospective experimental validations being more common than prospective ones. Moreover, there are many unexplored areas where AI could be applied, such as in salt formation processes. CFD deals with millions of discrete points, real-world implementation relies heavily on access to high-performance computing resources. Thus, advancements in GPU technology and parallel processing are critical to enabling practical, real-time CFD-AI applications in pharmaceutical formulation development [151]. Proficiency in fluid dynamics, simulation software, and data interpretation necessitates specialized workforce training. Developing interdisciplinary training programmes can bridge this gap, ensuring effective adoption and implementation of CFD in real-world industrial settings. While significant progress has been made for designing advanced drug delivery nanocarriers, challenges remain in modeling complex nanoparticles, particularly those with high surface charges or intricate structures. Such particles often exhibit unique behaviors during formation, particle-to-particle interactions, and formulation stability, necessitating specialized models. For instance, nanoparticles with high surface charges may experience strong electrostatic interactions, affecting their aggregation and stability. Traditional physics-based models, like molecular dynamics (MD), can provide atomic-level insights but may struggle with larger-scale phenomena. Coarse-grained (CG) methods or dissipative particle dynamics (DPD) can bridge this gap but may lack precision in capturing charge-specific effects. Data-driven models, such as ML can uncover hidden patterns in large datasets but often lack interpretability. Hybrid models combining quantum mechanics for electronic properties and MD for structural dynamics, supplemented by ML for predictive analytics, could offer a comprehensive solution. Future research should focus on developing specialized models tailored to high-charge nanoparticles, ensuring accurate predictions of their behavior in biological environments. This will enhance the design of stable, efficient NCs, advancing personalized medicine and targeted therapies.

Algorithmic bias in ANN models, often stemming from imbalanced training data or flawed model architecture, reflects a broader lack of ethical scrutiny in AI development. To align with principles such as fairness, transparency, and data privacy, the integration of SHapley Additive exPlanations (SHAP), and federated learning (FL) is critical. SHAP enhances explainability and supports systematic evaluation of bias, while ensuring that interpretability does not compromise the privacy protections essential to responsible AI implementation while FL allows multiple entities to collaboratively train a model while keeping the data decentralized [152, 153]. The green AI strategies like sparsity in neural networks, quantization, and low-precision arithmetic are required to reduce computational load. Tools including CarbonTracker and CodeCarbon should be integrated with the models to quantify energy use, enabling more sustainable AI practices [154]. Beyond operational efficiency, AI adoption aligns with global sustainability initiatives. Case studies in lyophilization and HVAC systems demonstrate that AI-driven optimizations can reduce energy use by up to 75% and cut CO₂ emissions by metric tons annually, addressing both economic and environmental imperatives. Future work should expand these applications to other high-energy processes. Expanding the use of AI in pharmaceutical liquid dosage formulations presents both significant challenges and promising opportunities for the pharmaceutical research community.

## Conclusion

This article explores the application of AI tools in the development of liquid dosage formulations. By comparing AI-based strategies with traditional trial-and-error methods, which are labor-intensive and time-consuming, it highlights the advantages of AI in accelerating the formulation process. AI enables pharmaceutical companies to generate cost-effective predictions more efficiently. The article discusses various AI algorithms tailored for different objectives and provides general guidance on model selection. It concludes that AI tools have been applied across several key stages of liquid dosage manufacturing, though real-time application remains limited. When you automate as much of a task as possible, we can spend our effort on the task’s machines cannot do yet. Each work that we hand over to the authority of technology frees up time and energy to pour into the next stage of growth. The study also identifies potential future directions and research gaps, suggesting that AI could significantly contribute to the development of smart, autonomous pharmaceutical manufacturing processes in the near future. While this review highlights AI’s transformative role in manufacturing efficiency, future work could explore its integration with PK/PD modeling to co-optimize therapeutic efficacy. This would likely enhance production speed, reduce costs, and minimize waste, thereby lowering environmental impact and facilitating automation.

## Data Availability

No datasets were generated or analysed during the current study.
